# Economic purification of recombinant uricase by artificial oil bodies

**DOI:** 10.1186/s40643-022-00501-x

**Published:** 2022-02-06

**Authors:** Fatemeh Saadat, Peter Macheroux, Houshang Alizadeh, Seyed Hadi Razavi

**Affiliations:** 1 Independent Department of Biotechnology, College of Agriculture and Natural Resources, University of Tehran, Karaj, Iran; 2 Institute of Biochemistry, Graz University of Technology, Graz, Austria; 3 Department of Agronomy and Plant Breeding, University of Tehran, Karaj, Iran; 4 Department of Food Science & Technology, College of Agriculture & Natural Resources, University of Tehran, Karaj, Iran

**Keywords:** Caleosin, Downstream processing, Intein, Nanoemulsion, Triacylglycerol, Urate oxidase

## Abstract

Rasburicase is an expensive treatment used to control hyperuricemia caused by tumour lysis syndrome (TLS). In this study, a non-chromatographic method was designed based on nano-oil bodies for convenient and economical purification of the recombinant uricase. For this purpose, two chimaeras were synthesized with a different arrangement of the uricase, caleosin and intein fragments. After confirming the protein expression by measuring the uricase activity at 293 nm, purification was conducted through oil-body construction. The results were resolved on the 12% SDS-PAGE gel. Finally, the stability of the oil bodies was examined against different salts, surfactants, temperatures, and pH values. According to our results, the overexpression of uricase–caleosin chimaera under the T7 promoter in *Escherichia coli* led to the production of soluble protein, which was successfully purified by artificial oil bodies. The active uricase was subsequently released through the self-splicing of intein. Further investigations highlighted the importance of the free C-terminus of caleosin in constructing artificial oil bodies. Moreover, surfactants and low temperature, in contrast to salts, improved the stability of oil bodies. In conclusion, caleosins are an efficient purification tag reducing the cost of purification compared to conventional chromatography methods.

**Graphical Abstract:**

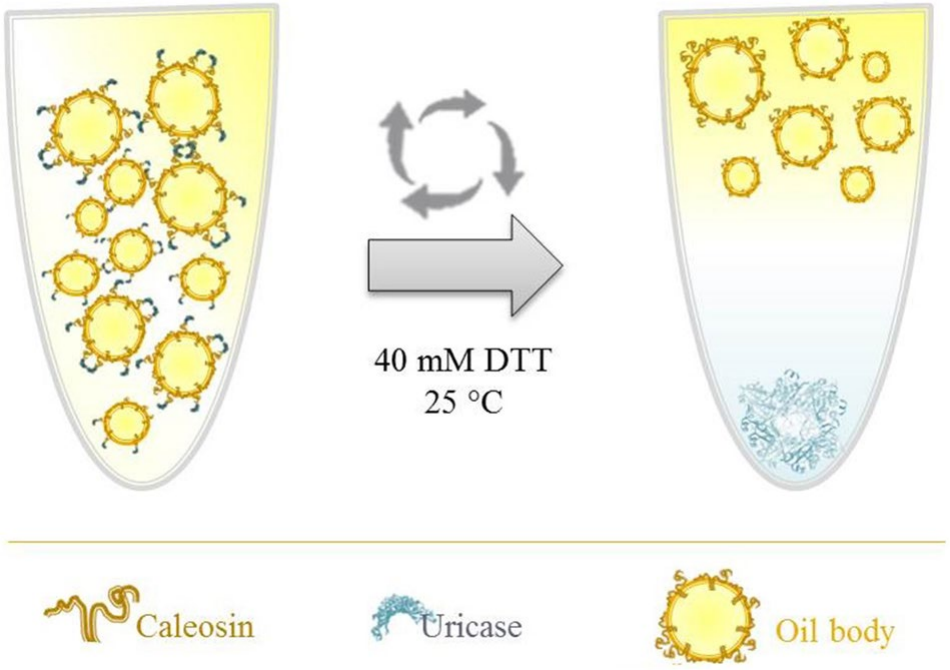

## Introduction

Uricase or urate oxidase (EC 1.7.3.3) is a homotetrameric enzyme that catalyses the oxidation of uric acid during purine catabolism. In contrast to most living organisms, the gene encoding the enzyme has become dysfunctional in the human lineage owing to some nonsense mutations (Kratzer et al. 2014). Although several advantages have been suggested for the evolutionary removal of uricase in humans, excessive uric acid levels are associated with the risk of hyperuricemia, gout, high blood pressure, metabolic syndrome, diabetes as well as heart and kidney diseases (Álvarez-Lario and Macarrón-Vicente 2010). Hyperuricemia is also a part of tumour lysis syndrome (TLS) that can be fatal in one-third of patients (Mayne et al. 2008). Therefore, a recombinant uricase from *Aspergillus flavus*, termed rasburicase, has been approved by the *Food and Drug Administration* (FDA) to control uric acid levels. However, treatment with this drug is expensive—as much as 7500 $ per day for a 70 kg person. Therefore, approaches to reduce the cost of treatment are highly desirable. According to economic analysis, the purification of uricase using an aqueous two-phase system (ATPS) instead of chromatography could save about 4000 $ per gram by reducing capital, consumables, and labour (Torres-Acosta et al. 2016).

ATPS has been known for the separation and concentration of cell organelles, proteins, nucleic acids, and low molecular weight compounds since the early 1960s (Raja et al. 2012). This water-in-water (W/W) emulsion is usually prepared based on the incompatibility of two water-soluble components, e.g. polymers, kosmotropic salts, alcohols and/or surfactants. Although ATPS is easier and faster than chromatography, the material costs are still remarkable. Moreover, solvent recycling and unclear separation mechanisms are among the main barriers (Pereira et al. 2020). Despite higher viscosity and interfacial tensions of oil-in-water (O/W) emulsions, they lack the drawbacks of ATPS. Furthermore, O/W emulsion droplets are used for various applications, such as drug delivery and bioavailability, encapsulation, and the food industry (Chao and Shum 2020).

Oil bodies are naturally formed using neutral lipids, phospholipids, and membrane proteins. The latter component constitutes the smallest part; however, that is the most critical in terms of the construction and stability of oil bodies. The amphiphilic nature of proteins allows them to coat oil particles, reduce the interfacial tension, and stabilize dispersions (Lam and Nickerson 2013). Oleosin and caleosin are the two best-known structural proteins of oil bodies (Purkrtová et al. 2015). Since oleosins were discovered about 30 years earlier than caleosins, more investigations have applied them for protein purification (Chiang et al. 2005, 2007; Liu et al. 2008; Choi and Chang 2009), protein targeting (Karg and Kallio 2009; Montesinos et al. 2016; Huang et al. 2017), bioencapsulation (Chang et al. 2013), enzyme immobilization (Chiang et al. 2006), and antibody production (Tseng et al. 2011). However, caleosins have the advantage to make smaller oil bodies, which are more useful in drug delivery (Chiang et al. 2011, 2012) and bioavailability (Chen et al. 2005). Owing to these advantages, we have thus focused on the purification of recombinant uricase using the nano-oil bodies containing caleosin instead of oleosin.

## Material and methods

### Construction of expression plasmids

The full-length sequence of uricase–intein–caleosin (UC) was custom-synthesized (GeneralBiosystems, U.S.A.) and cloned into the pET28α vector (University of Tehran, Iran). The pET-UC plasmid template was used to synthesize the caleosin–intein–uricase chimaera (CU) by overlap extension (OE)-PCR through three steps. In the first step, each fragment was amplified separately using the primers listed in Table 1. The oligonucleotides were purchased from SinaColon (Iran) and Bioneer Inc. (South Korea). The produced intein harboured overlapping sequences for uricase and caleosin at its C and N-terminus, respectively. Then, an equimolar amount of all three fragments was mixed for assembling the CU chimaera. Finally, the OE-PCR product was amplified using the oligonucleotide forward primer of caleosin (FC) and the reverse primer of uricase (RU). All the mentioned reactions were performed with Phusion high-fidelity DNA polymerase (Fermentas) using GeneAmp PCR System 9700 in the following program: pre-denaturing (94 °C, 3 min), 25 cycles of denaturing (94 °C, 40 s), annealing, (50 °C, 30 s), and extension (72 °C, 1 min/kb), and a final extension (72 °C, 10 min). The desired PCR product was cloned into the pJET1.2/blunt and then digested with NotI and EcoRI to ligate into the expression vector pET28α. The accuracy of the recombinant constructs was confirmed by digestion and DNA sequence analysis (Microsynth, Austria) using the T7 sequencing primers.

**Table 1:** List of primer sequences used in this study

Primer name	Nucleotide sequence
FU	GAATTCAAAATGTCTGCAGTAAAG
RU	GCGGCCGCATTATAACTTGGATTTCAAGGAAGA
FC	GAATTCAAAATGGGATCAGAGATCGACGATTC
RC	GCGGCCGCATTATCTACC
FIC	CTAAGATGAACATGGGTAGAGCCGTATCAGGTGATACTATCGTAATG
RIU	CTAGCGGCCTTTACTGCAGAATTATGGACAATGAATCCGTTG

### Bacterial strains and expression method

The resulting plasmids were transferred into *Escherichia coli* Top10 and BL21 (DE3) for the recombinant construct amplification and expression, respectively. The cells were grown in LB medium containing 50 µg ml^−1^ kanamycin. Expression was optimized according to the Sambrook protocol (Sambrook et al. 1989) at a final OD_600_ of 0.7. Then, the cells were harvested by centrifugation (4500×*g*, 4 °C, 20 min). The pellet was re-suspended in 3% of the initial LB volume of phosphate buffered saline (pH 8.5, 0.137 M NaCl, 0.027 M KCl, 0.01 M Na_2_HPO_4_, and 0.0018 M KH_2_PO_4_), and then lysed on ice by sonication using a LABSONIC P (Sartorius AG, Germany). Insoluble material was pelleted by centrifugation, and the soluble fraction was stored at − 20 °C for further analyses.

### Protein purification

The procedure for protein purification was carried out as described by Tai et al. (Tai et al. 2002). In brief, 250 μl of the extracted proteins, 150 μg phosphatidylcholine (Sigma-Aldrich) and 15 mg olive oil were suspended in 1 ml of 0.1 M sodium phosphate buffer (pH 7.5). After mixing, three times sonication with 30% amplitude was done on ice for 20 s for the constitution of artificial oil bodies (AOBs). After centrifugation (10,000×*g*, 4 °C, 15 min), the AOBs were collected and subjected to the desired pH or 40 mM DTT for 16 h to release the target protein. Finally, the oil and aqueous phases were separated by centrifugation and each phase was analysed by SDS-PAGE and enzyme assay.

### Protein purity and molecular weight determination

The purity of the proteins was determined on 12% of sodium dodecyl sulfate-polyacrylamide gel electrophoresis (SDS-PAGE). Gel electrophoresis was performed using Mini-PROTEAN Tetra Cell (BioRad) and proteins were stained with Coomassie blue R-250. A prestained protein ladder (ThermoFisher, 26616) was used to determine the molecular weight of the proteins.

### Microscopy of AOBs

The size and shape of AOBs constituted with and without the chimaera proteins were observed under a light microscope (Zeiss Axioplan 2, Germany). The result was interpreted by ImageJ software (version 1.8.0) (Schneider et al. 2012).

### AOBs stability test

The AOB’s stability was measured by turbidity changes at 600 nm at room temperature (Chen et al. 2004). One millilitre of suspension mixture was placed in a disposable cuvette and treated with different parameters, including salts, surfactants, pH, and temperature.

### Uricase activity assay

The uricase activity was determined by continuous spectrophotometry. The reaction solution contained 0.11 mM uric acid (Carl Roth) prepared in 20 mM boric acid buffer pH 9. The enzymatic reaction was monitored at 293 nm for 5 min using a SPECORD 205 Analytik Jena (Germany). Subsequently, the activity was calculated using the Lambert–Beer law (extinction coefficient at 293 nm = 12.6 mM^−1^ cm^−1^). A unit (U) of uricase activity was defined as the consumption of one micromole of uric acid per min at pH 9.0 at 25 °C. Finally, the protein concentration was measured using Bradford assay (Bradford 1976) for calculating the specific activity.

### Molecular modelling and docking

The intensive mode of Phyre2 (www.sbg.bio.ic.ac.uk/phyre2) was utilized to predict the 3D structure of the chimaera proteins (Kelley et al. 2015). The c1r56H from protein data bank (PDB) was used for retrieving the uricase domain. Besides, 17 structures (including c3u0kA, c4i2yB, c2hpkA, d1dtla, c3evrA, c3sibA, c3dtpF, d1iq3a, and c3ek7A for caleosin, and c4o1rA, c2imzA, c2in0A, d1am2a, d1mi8a, c2keqA, c4o1sB, and c2jmzA for intein) were selected on the basis of sequence identity (> 88%) for the modelling of caleosin and intein. Subsequently, the created models were refined and verified using ModRefiner (https://zhanglab.ccmb.med.umich.edu/ModRefiner/) and SAVES v5.0 (http://servicesn.mbi.ucla.edu/SAVES/), respectively (Colovos and Yeates 1993; Xu and Zhang 2011). Then, molecular docking was applied to predict the binding of modelled chimaeras against uric acid by SwissDock (www.swissdock.ch) (Grosdidier et al. 2011). A grid box of 30 points in all directions of L147 was defined with a flexibility of 3 Å. Finally, the outputs were compared by the free energy of ligand binding (ΔGbinding, kcal/mol). The macromolecular structures were visualized by BIOVIA Discovery Studio (version 3.5).

### Statistical analysis

Each experiment was performed in three replications using freshly prepared samples. The collected data were analysed using R (version 3.6.3). The one-way analysis of variance (ANOVA) and two-sample Wilcoxon test was used to compare the results, and *p* < 0.05 was considered a significant variation.

## Results and discussion

### Expression of UC and CU chimaeras in *E. coli*

The UC and CU chimaeras were successfully constructed and transformed into *E. coli* BL21. To determine the optimum expression conditions, the effect of temperature and inducer concentration were investigated. The best result was obtained with 1 mM IPTG at 20 °C and overnight incubation.

**Fig. 1:**
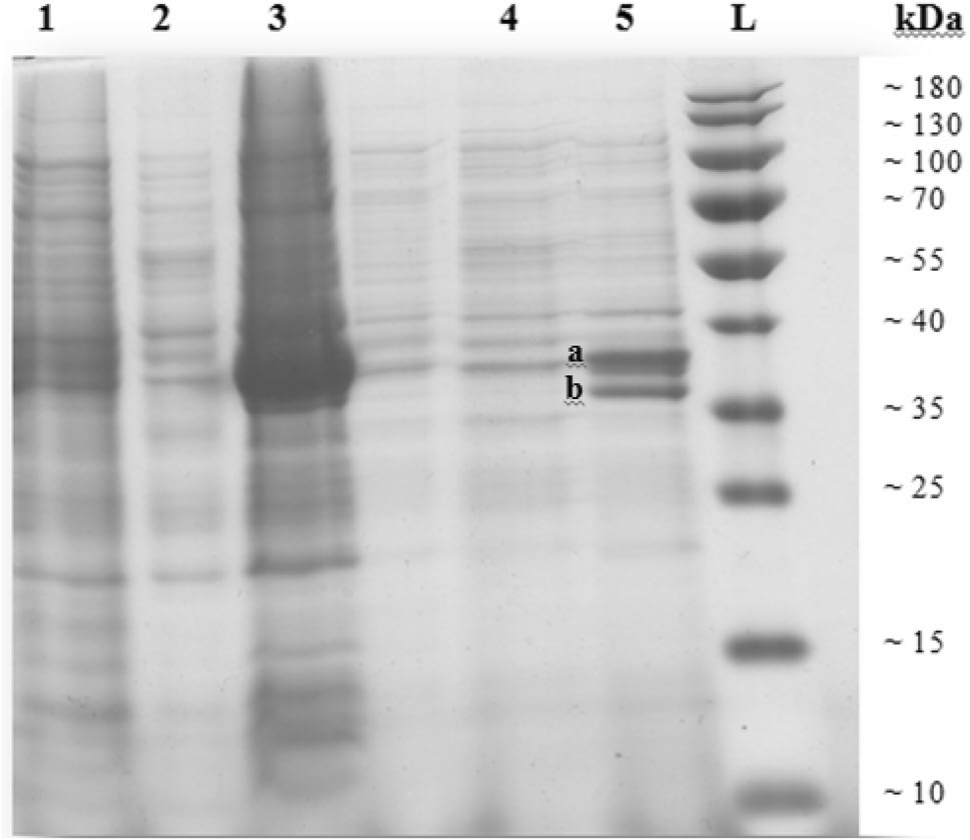
SDS-PAGE of UC chimaera expressed in *E. coli* with 1 mM IPTG and overnight incubation at 20 °C. Soluble (1) and insoluble (2) fractions before IPTG induction and soluble (3) and insoluble (4) fractions after induction were resolved on SDS-PAGE. Lane 5 is a dilution of lane 3. The positions of intein–caleosin (~ 40 kDa) and uricase (~ 34 kDa) were indicated by a and b, respectively

The protein content of induced and non-induced recombinant strains was analysed using SDS-PAGE. Accordingly, the production of recombinant protein under the control of the T7 promoter led to the appearance of a sharp band in the soluble fraction of the cell lysate (Fig. 1). Although a molecular mass of 72 kDa was expected for both chimaeras, the expressed proteins exhibited a mobility corresponding to a molecular mass between 35 and 40 kDa. The analyses of the diluted sample revealed two distinct bands for uricase (~ 34 kDa) and intein–caleosin (~ 40 kDa). Subsequently, the activity of the produced proteins was confirmed by uricase assay. Apparently, cleavage of the fusion protein has occurred under the reducing conditions used for SDS-PAGE. The faster migration of intein–caleosin could be caused by the binding of caleosin to available Ca^2+^ (Chen et al. 1999). In contrast, the multi-subunit structure of uricase is supposed to show less mobility on SDS-PAGE since they are resistant to denaturation by SDS (Pitts et al. 1974).

It is noteworthy that membrane proteins like caleosins are insoluble and get stuck in the pellet. However, the chimaera proteins were soluble.

### Purification of uricase by AOB system

**Fig. 2:**
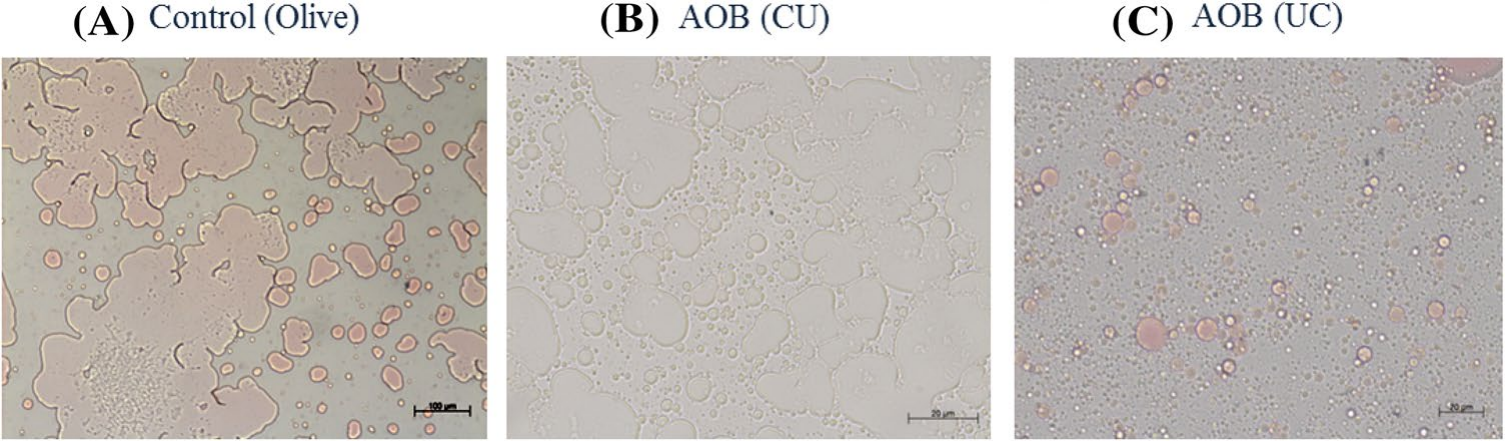
Light microscopy of artificial oil bodies constituted with and without chimaera proteins. **A** Represents sonicated olive oil with no chimaera protein, while **B** and **C** show the sonicated olive oil in the presence of CU and UC chimaera, respectively. The bar represents 100 μm for control and 20 μm for two other

The purification of recombinant uricase was conducted using the AOB-based system. In the first step, AOBs were successfully formed using oil, phosphatidylcholine, and soluble fraction of cell lysate (with and without the chimaera proteins). The construction of AOBs was confirmed by microscopic visualization. As can be seen in Fig. 2, the AOBs were almost coalescence in the control condition, which was without the chimaera proteins. However, the presence of UC protein makes the nanoparticles (about 0.2 μm) (Fig. 2C). The comparison between the chimaera proteins indicates that CU is not a competent candidate for the construction of AOBs (Fig. 2B). It seems a free C-terminus might be required for caleosin to build more stable and smaller droplets. However, it has been reported that the N-terminus is also necessary for targeting caleosin to oil bodies (Purkrtová et al. 2015).

**Fig. 3:**
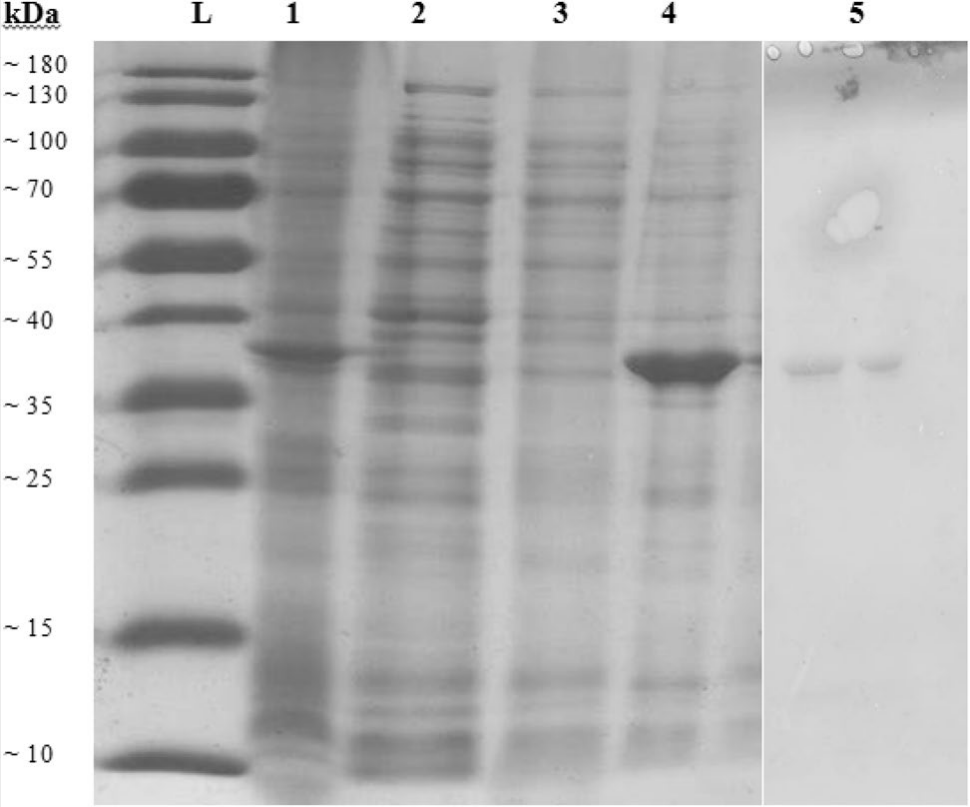
SDS-PAGE of purified uricase through AOB system. Three fractions including, AOB (lane 1), aqueous phase (lane 2 and 3), and soluble uricase, were resolved on SDS-PAGE. The purified uricase is represented after the first cycle of purification (lane 4) and after three times washing (lane 5)

After centrifugation, the AOBs separated from the aqueous phase and washed to eliminate the non-specific proteins (Fig. 3, lanes 2 and 3). Then, uricase was released by inducing the self-cleaving intein through shifting the pH and/or using 40 mM DTT at room temperature. Accordingly, uricase was retrieved in the aqueous phase (Fig. 3, lane 4), whereas intein–caleosin (Fig. 3, lane 1) remained in AOBs. Finally, all the mentioned fractions were resolved on SDS-PAGE (Fig. 3). Repetition of purification steps improved the purity of the uricase (Fig. 3, lane 5).

The specific activity of the enzyme was increased from 0.53 U/mg in the crude extract to 15.2 U/mg in the purified enzyme, which is comparable with the purified uricases by chromatography columns (Fazel et al. 2014; Khaleghi and Asad 2021).

### AOB stability

The AOB particles are shaped by balancing the attractive and repulsive forces of structural proteins (Tzen et al. 1992). Therefore, any change in pH, ions or solvent that causes protein unfolding affects emulsion stability.

Our investigations on a wide range of pH values (3 to 11) revealed phase separation at the pH around the isoelectric point (pI) of caleosin (pH 5). Indeed, a gradual increase in the emulsifying property occurs as the pH value gets far from the isoelectric point (Wang et al. 2019; Gao et al. 2021). Moreover, the AOB droplets aggregate at pH 3 to 4 since the acidic environment increases surface hydrophobicity of oil bodies and thus leads to coalescence (Gao et al. 2021).

**Fig. 4:**
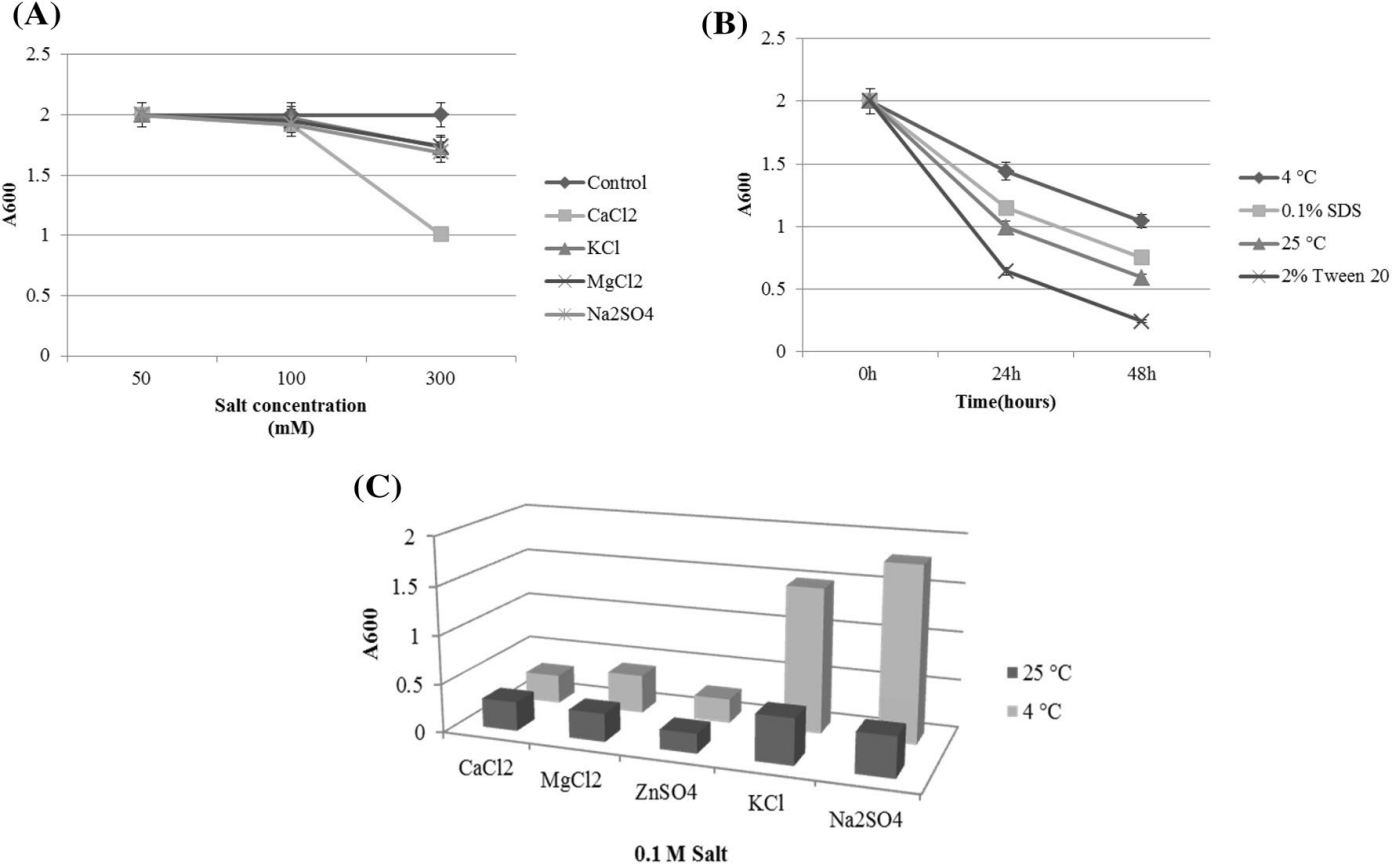
Turbidity tests of AOBs at 600 nm. The measurements were conducted at room temperature and pH 7.5 unless mentioned. The constituted oil bodies were exposed to **A** salt concentrations (50–300 mM) for 15 min, **B** temperature and surfactants for 24 h and **C** 0.1 M monovalent (NaCl and KCl) and divalent (MgCl_2_, CaCl_2_, and ZnSO_4_) salts for 24 h at 4 °C and 25 °C

The effects of three concentrations of 50, 100, and 300 mM of different salts, including MgCl_2_, CaCl_2_, KCl, and Na_2_SO_4_, were also traced on AOB suspension for 15 min by turbidity tests. All the salts caused instability proportional to the ionic strength (Fig. 4A). However, the rapid reduction in turbidity by 0.3 M CaCl_2_ (*p* < 0.05) could occur as a result of the interaction of calcium with the EF_hand motif placed on the N-terminal domain of caleosin. This Ca^2+^-binding motif responds to biotic and abiotic stresses and plays a role in releasing triacylglycerols from oil bodies during seed germination (Poxleitner et al. 2006; Partridge and Murphy 2009; Shimada and Hara-Nishimura 2010). Although calcium caused sedimentation of AOBs, its low concentrations (7.5 mM) have been used as a divalent to cross-link oil-body proteins and Pickering stabilizing (Liu et al. 2017). It has been reported that the Pickering emulsions need an oil volume fraction (*φ*) of greater than 0.2 (Guo et al. 2021).

In contrast to salts, surfactants are generally considered to act as emulsifying agents because of reducing surface tension, breaking hydrophobic interactions of proteins and increasing elasticity, viscosity, and electronegative repulsion (Sukhotu et al. 2014). Although using 2% Tween 20 had no positive effect on AOB stability, adding 0.1% SDS rendered the suspension more stable (Fig. 4B).

A comparison between the stability of AOBs at 4 °C and room temperature showed that the lower temperatures prevent the emulsion coalescence (Fig. 4B), even if adding 0.1 M salts (Fig. 4C). However, sensitivity to divalent cations (Ca^2+^, Mg^2+^, and Zn^2+^) is not affected by temperature changes (Fig. 4C). The same results have been reported for emulsions containing calcium or magnesium (Ramkumar et al. 2000; Romero-Guzmán et al. 2020). Our further investigations indicated that AOBs without the chimaera protein also show the same instability towards divalent salts (data not shown). Therefore, the reason for emulsion instability differs depending on the ionic strength and oil volume fraction.

### Effect of C- and N-terminal fusion on uricase activity

**Fig. 5:**
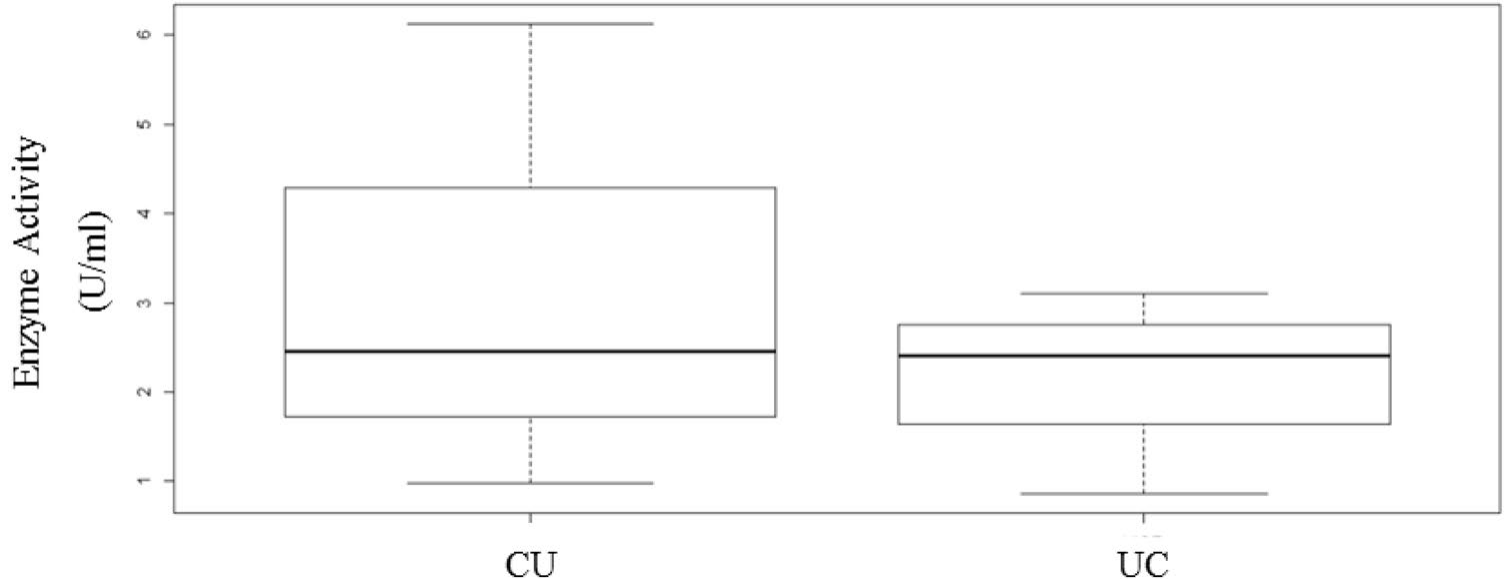
Comparison of uricase activity of CU and UC chimaeras to investigate the effects of tag on uricase activity. A non-parametric two-sample Wilcoxon test was used to compare the results (*p* < 0.05)

Two chimaeras, CU and UC, were designed to study the effect of C- and N-terminal fusion on uricase activity. As shown in Fig. 5, the enzyme activity was lower in UC chimaeras (*p* < 0.05).

**Fig. 6:**
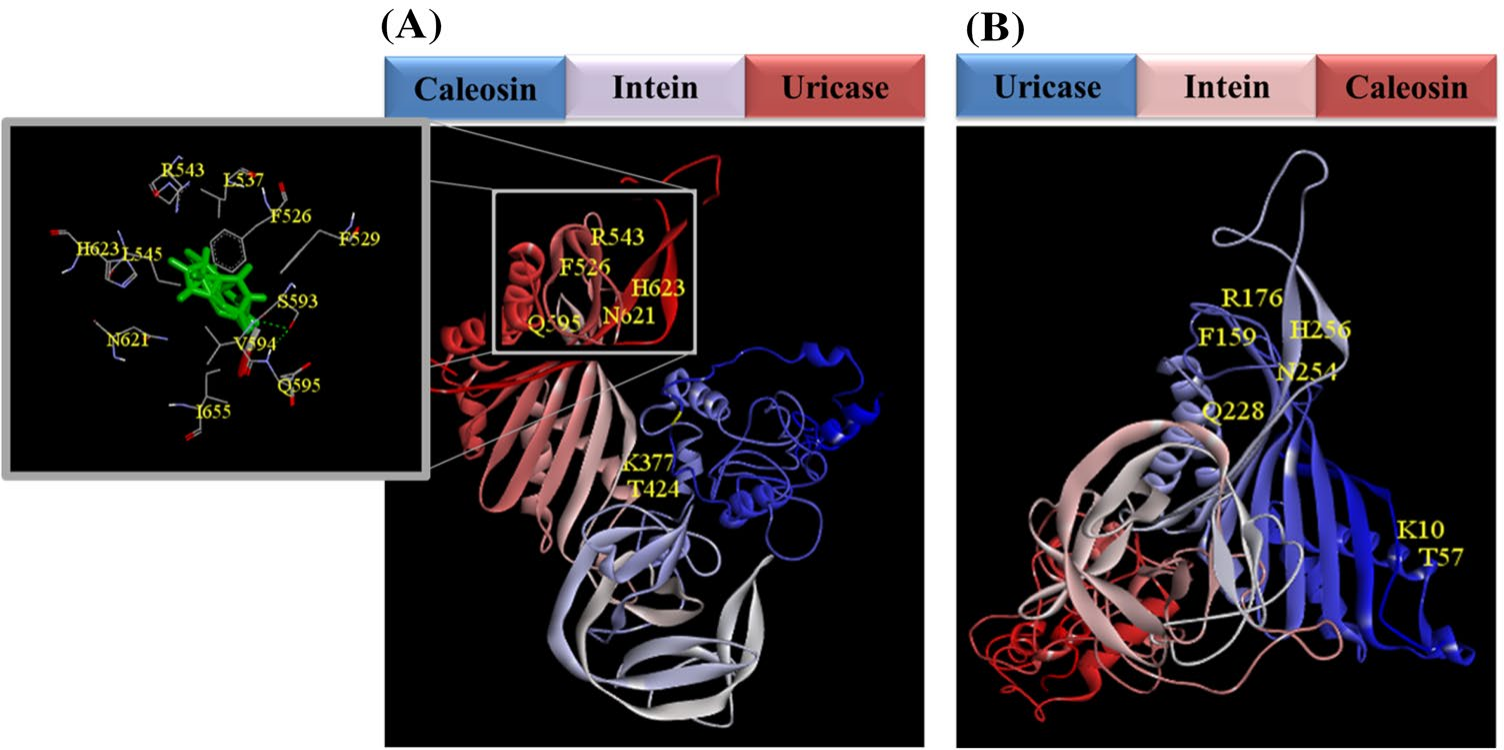
The predicted structure for **A** CU and **B** UC chimaeras by Phyre2. The domain arrangements with the corresponding colours are shown on top of the shapes. The docking complex between CU chimaera and uric acid is also magnified

To evaluate the binding affinity of the chimaera proteins to the uric acid, the 3D structure was modelled using Phyre2. Subsequently, 88% and 92% of residues of CU and UC were represented at > 90% confidence (Fig. 6). Furthermore, a docking study by default parameters of SwissDock revealed no binding at the expected points of UC chimaera. However, several binding sites were predicted for CU chimaera, and one of them included the expected residues (Δ*G* = − 6.97 kcal/mol). Indeed, uricase consists of 301 residues in which the catalytic triad (T57* K10* H256) were delimited by the six conserved residues (R176-Q228, N254-T57, and F159) (Gabison et al. 2008). As it is shown in Fig. 6, most of the defined residues (except T57* K10*) were identified on CU chimaera.

## Conclusions

Protein purification with a history of over 200 years is still one of the main challenges facing scientists during the production and study of proteins. Although chromatography is one of the most commonly used approaches for protein purification, it is associated with some drawbacks, including high cost, time-consuming and scale limitation. Compared to chromatography, the AOB system is an easy, fast and inexpensive method that is feasible for recombinant protein purification.

Although protein purification using oil bodies has only been reported for oleosin, we selected caleosin as an efficient tool in constructing nano-oil bodies (Chen et al. 2004). In addition to convenient purification, stability and high surface absorption of the nano-oil bodies make them useful for immobilization and drug delivery of recombinant proteins like uricase. Moreover, constituted oil bodies can be used as biocompatible and renewable emulsifiers in the pharmaceuticals, cosmetics, and food industries to retrieve other parts of production costs. Therefore, our procedure would be more cost-effective than conventional chromatography methods.

## Data Availability

All data generated or analysed during this study are included in this published article.

## Abbreviations

AOB: Artificial oil bodies
ATPS: Aqueous two-phase system
FDA: Food and Drug Administration
UC: Uricase–intein–caleosin chimaera
CU: Caleosin–intein–uricase chimaera
